# Partial recovery of peripheral blood monocyte subsets in head and neck squamous cell carcinoma patients upon radio(chemo)therapy is associated with decreased plasma CXCL11

**DOI:** 10.1186/s12885-024-12177-x

**Published:** 2024-04-12

**Authors:** Christian Idel, Jonas Fleckner, Kirstin Plötze-Martin, Lotte Werner, Dirk Rades, Marie-Nicole Theodoraki, Linda Hofmann, Diana Huber, Anke Leichtle, Thomas K. Hoffmann, Karl-Ludwig Bruchhage, Ralph Pries

**Affiliations:** 1 Department of Otorhinolaryngology and Head & Neck Surgery, University of Luebeck, Luebeck, 23538 Germany; 2 Department of Radiation Oncology, University of Luebeck, Luebeck, 23538 Germany; 3 Department of Otorhinolaryngology and Head & Neck Surgery, Ulm University Medical Center, Ulm, 89075 Germany; 4 Department of Otorhinolaryngology, Technical University Munich, Munich, Germany

**Keywords:** Head and Neck squamous cell carcinoma, Radio(chemo)therapy, Monocyte subsets, PD-L1, Adhesion molecules, CXCL11

## Abstract

**Background:**

Head and neck squamous cell carcinoma (HNSCC) represents a common and heterogeneous malignancy of the oral cavity, pharynx and larynx. Surgery and radio(chemo)therapy are the standard treatment options and also have great influence on the composition of the tumor microenvironment and immune cell functions. However, the impact of radio(chemo)therapy on the distribution and characteristics of circulating monocyte subsets in HNSCC are not fully understood.

**Methods:**

Expression patterns of adhesion molecules and chemokine receptors CD11a (integrin-α L; LFA-1), CD11b (integrin-α M; Mac-1), CD11c (integrin-α X), CX3CR1 (CX3CL1 receptor) and checkpoint molecule PD-L1 (programmed cell death ligand-1) were investigated upon radio(chemo)therapeutic treatment using flow cytometry. Furthermore, comprehensive analysis of plasma cytokines was performed before and after treatment using ELISA measurements.

**Results:**

Our data reveal a partial recovery of circulating monocytes in HNSCC patients upon radio(chemo)therapeutic treatment, with differential effects of the individual therapy regimen. PD-L1 expression on non-classical monocytes significantly correlates with the individual plasma levels of chemokine CXCL11 (C-X-C motif chemokine 11).

**Conclusions:**

Further comprehensive investigations on larger patient cohorts are required to elucidate the meaningfulness of peripheral blood monocyte subsets and chemokine CXCL11 as potential bioliquid indicators in HNSCC with regard to therapy response and the individual immunological situation.

## Introduction

Head and neck squamous cell carcinoma (HNSCC) is a common tumor entity of the oral cavity, pharynx, larynx and paranasal sinuses with a poor prognosis [1, 2]. Besides surgery, radiation therapy with or without concomitant chemotherapy are currently the primary standard therapeutic options for HNSCC patients [3, 4].

Furthermore, research advancements in tumor biology led to the development of novel promising approaches such as immune checkpoint inhibitors (ICIs) of checkpoint molecules PD-1 (programmed death 1) and PD-L1 (programmed death ligand 1) in order to prevent T-cell anergy and exhaustion [5–7]. However, it is well known that radio(chemo)therapy often fails due to different immunostimulatory as well as immunosuppressive effects [8]. For example, dying tumor cells in response to radiation may stimulate anti-tumor activities of different immune cells such as dendritic cells or cytotoxic T-cells [9, 10], but radiotherapy can also result in a counteracting myelosuppression, which is defined as a decrease in the ability of the bone marrow to produce blood cells and which is also the major dose-limiting factor of chemotherapy [11, 12]. These differential immunological consequences of radio(chemo)therapeutic treatment are not fully understood and need to be investigated more thoroughly.

Especially cells from the monocytic differentiation line are known to be important regulators of cancer development and progression [13]. In this context, radio(chemo)therapy has been shown to significantly enhance the proportion of rectal cancer-infiltrating CD8^+^ T cells and the percentage of tumor necrosis factor α (TNFα) producing monocytes [14]. It has recently been shown for locally advanced rectal cancer, that PD-L1 expression on monocytes suppresses the cell-mediated immunity and is inversely correlated with the individual response to preoperative radio(chemo)therapy [15].

However, a detailed understanding of the distribution of peripheral blood monocyte subsets and expression patterns of cytokines and proteins required for adhesion, invasion and immune regulation in HNSCC patients upon radio(chemo)therapeutic treatment remains incomplete.

Following the development in the bone marrow, monocytes circulate in the peripheral blood stream, and around three days later, they migrate to peripheral tissues, as a consequence of homeostasis and inflammation [16]. Circulating monocytes are capable of pro- as well as anti-tumor immune functions and act as progenitors of tumor infiltrating macrophages [13]. Monocytes can be classified into three different subpopulations based on their CD14 and CD16 expression, namely “classical” monocytes (CD14^++^CD16^-^), “intermediate” monocytes (CD14^+^CD16^+^) and “non-classical” monocytes (CD14^dim+^CD16^+^) [17–19]. All peripheral monocyte subsets are able to acquire macrophage morphology and characteristics, but the exact differentiation potential of the different subsets remains incomplete [20].

Our recent data revealed that increased percentages of circulating non-classical monocytes significantly correlated with elevated levels of overall monocytic PD-L1 in HNSCC patients, all of which were analyzed prior to any surgical or therapeutic treatment [21].

Aim of this study was to analyze the individual distribution of circulating monocyte subsets in HNSCC patients as well as associated expression levels of adhesion molecules and chemokine receptors CD11a (integrin-α L; LFA-1), CD11b (integrin-α M; Mac-1), CD11c (integrin-α X), CX3CR1 (CX3CL1 receptor) and checkpoint molecule PD-L1 (programmed cell death ligand-1) using flow cytometry, all of which are known to be differentially expressed in response to different environmental conditions [22]. Furthermore, comprehensive evaluation of expression patterns of plasma cytokines was carried out, in correlation with the immunological situation before and after radio(chemo)therapeutic treatment.

The study aimed to better understand the interplay between the individual treatment regimen and the peripheral immunologic consequences on circulating monocytes as a potential prognostic biomarker for therapy response assessment in patients with HNSCC.

## Results

### Monocyte subset distribution upon radio(chemo)therapy

**Fig. 1:**
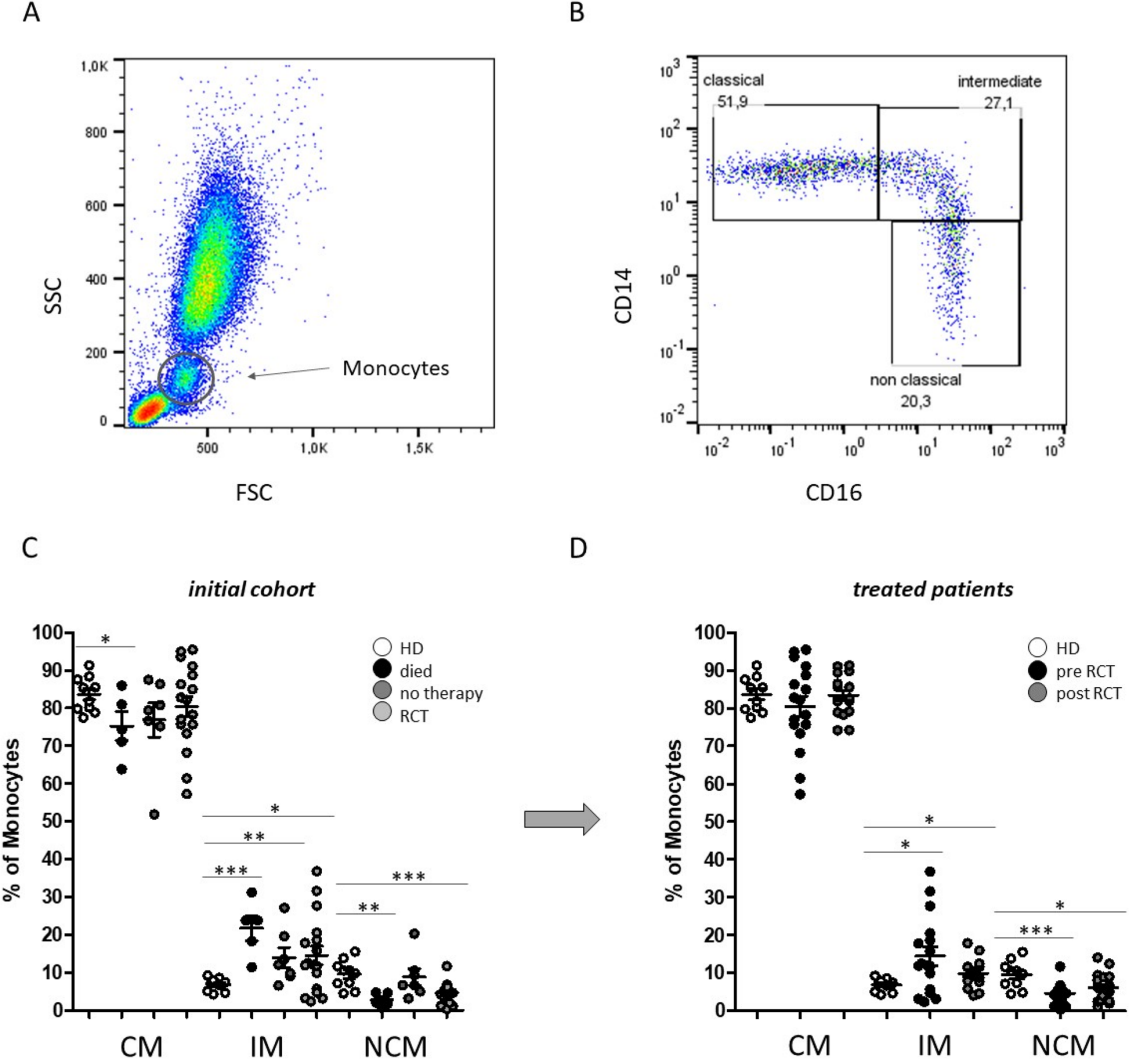
Flow cytometric analysis of peripheral blood monocyte subsets. **A** Representative example gating scheme of monocyte subset analysis with regard to the forward scatter (FSC)/sideward scatter (SSC) characteristics and the **B** CD14/CD16 expression levels. **C** Percentages of circulating classical (CM), intermediate (IM) and non-classical monocytes (NCM) in the peripheral blood of HNSCC patients that unfortunately ‘died’ during the course of treatment, patients that received ‘no therapy’ and patients that were able to receive radio(chemo)therapeutic treatment (RCT). **D** Monocyte subset abundances of HNSCC patients before (pre RCT) and after (post RCT) radio(chemo)therapeutic treatment compared to healthy donors (HD). *: *p* < 0.05; **: *p* < 0.01; ***: *p* < 0.001

Whole blood flow cytometric measurements were performed to investigate the individual abundances of peripheral blood monocyte subsets and associated expression levels of different adhesion molecules upon radio(chemo)therapy of patients with HNSCC. Gating of monocyte subsets was conducted as described previously [23]. In short, CD45 was used as a pan leukocyte marker to facilitate whole blood measurement and monocytes were first roughly gated by their FSC/SSC characteristics and the positivity for CD14 and CD16. Neutrophil granulocytes, NK-cells and B-cells were excluded by means of HLA-DR which is specific for monocytes. Remaining monocytes were then subgated into CD14^++^CD16^-^ (classical), CD14^++^CD16^+^ (intermediate) and CD14^dim+^CD16^+^ (non-classical) monocytes (Fig. 1).

From our initial cohort of 29 HNSCC patients, we were able to investigate 17 patients before and after about 6 month of radio(chemo)therapeutic treatment, because five patients unfortunately died during the course of treatment and seven patients’ condition did not allow radio(chemo)therapeutic treatment. Thus, first we subdivided our initial cohort into three groups (died, no therapy, therapy) with regard to the percentages of peripheral blood monocyte subsets. Data revealed significantly redistributed abundances of all three monocyte subsets in patients who unfortunately died during the course of treatment compared to healthy donors (Fig. 1C). These data of course have to be interpreted with caution due to different causes of dead, but nevertheless may add important information to an overall picture. Data of Results must be presented in with caution considering the limited sample size Moreover, significantly increased percentages of intermediate monocytes could be detected in the analysed cohorts compared to healthy donors (Fig. 1C). Furthermore, subset abundances of HNSCC patients before and after radio(chemo)therapeutic treatment were investigated. Our data revealed no overall significant differences between the pre and post treatment situation, but a smaller spread of percentage values of classical (CM) and intermediate (IM) monocytes upon radio(chemo)therapy (Fig. 1D).

### Monocytic adhesion molecules upon radio(chemo)therapy

**Fig. 2:**
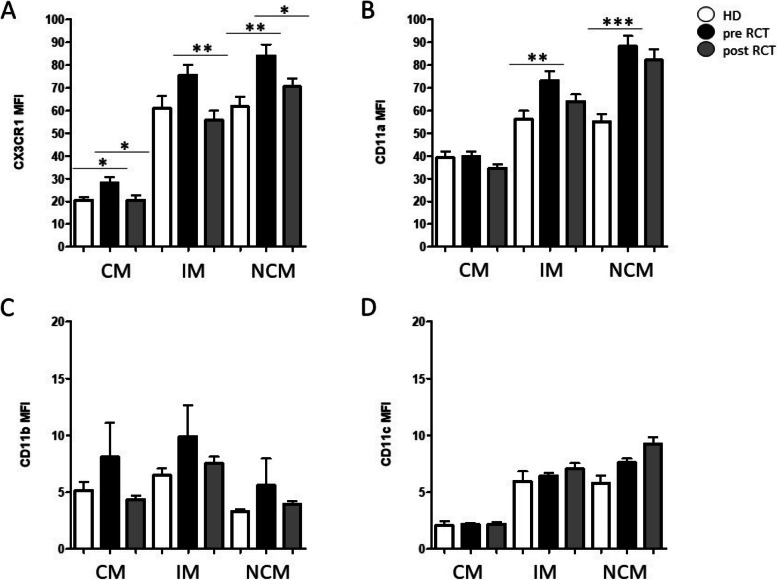
Adhesion molecules on peripheral blood monocyte subsets (CM: classical monocytes; IM: intermediate monocytes; NCM: non-classical monocytes) before and after radio(chemo)therapeutic treatment (RCT). Measurements revealed significant alterations of **A** CX3CR1 and **B** CD11a expression on intermediate and non classical monocytes prior to RCT treatment compared to healthy donors (HD), respectively. Measurements of adhesion molecules **C** CD11b and **D** CD11c revealed no significant differences. *: *p* < 0.05; **: *p* < 0.01; ***: *p* < 0.001. MFI: mean fluorescence intentsity

Expression levels of adhesion molecules and chemokine receptors CD11a (integrin-α L; LFA-1), CD11b (integrin-α M; Mac-1), CD11c (integrin-α X), and CX3CR1 (CX3CL1 receptor) on peripheral blood monocyte subsets from HNSCC patients before and after radio(chemo)therapeutic treatment were analyzed using flow cytometry and compared to healthy donors (Fig. 2).

Measurements revelaled significantly increased pre-therapeutic expression levels of adhesion molecule CX3CR1 on classical (*p* = 0.0351) and non-classical monocytes (*p* = 0.0047) compared to healthy donors and a significantly decreased expression in response to therapeutic treatment in all three subsets (CM: *p* = 0.0301; IM: *p* = 0.0038; NCM: *p* = 0.0347) (Fig. 2A). Increased pre-therapeutic expression levels were also found for adhesion molecule CD11a in intermediate (*p* = 0.0093) and non-classical monocytes (*p* < 0.001) (Fig. 2B). Overall, expression levels of adhesion molecules CD11b and CD11c revealed no significant differences with regard to the analyzed conditions, whereas elevated levels of CD11b could be observed in all monocyte subsets before therapeutic treatment compared to healthy donors and the after treatment situation by tendency (Fig. 2C, D).

### Plasma cytokines of HNSCC patients upon radio(chemo)therapy

**Fig. 3:**
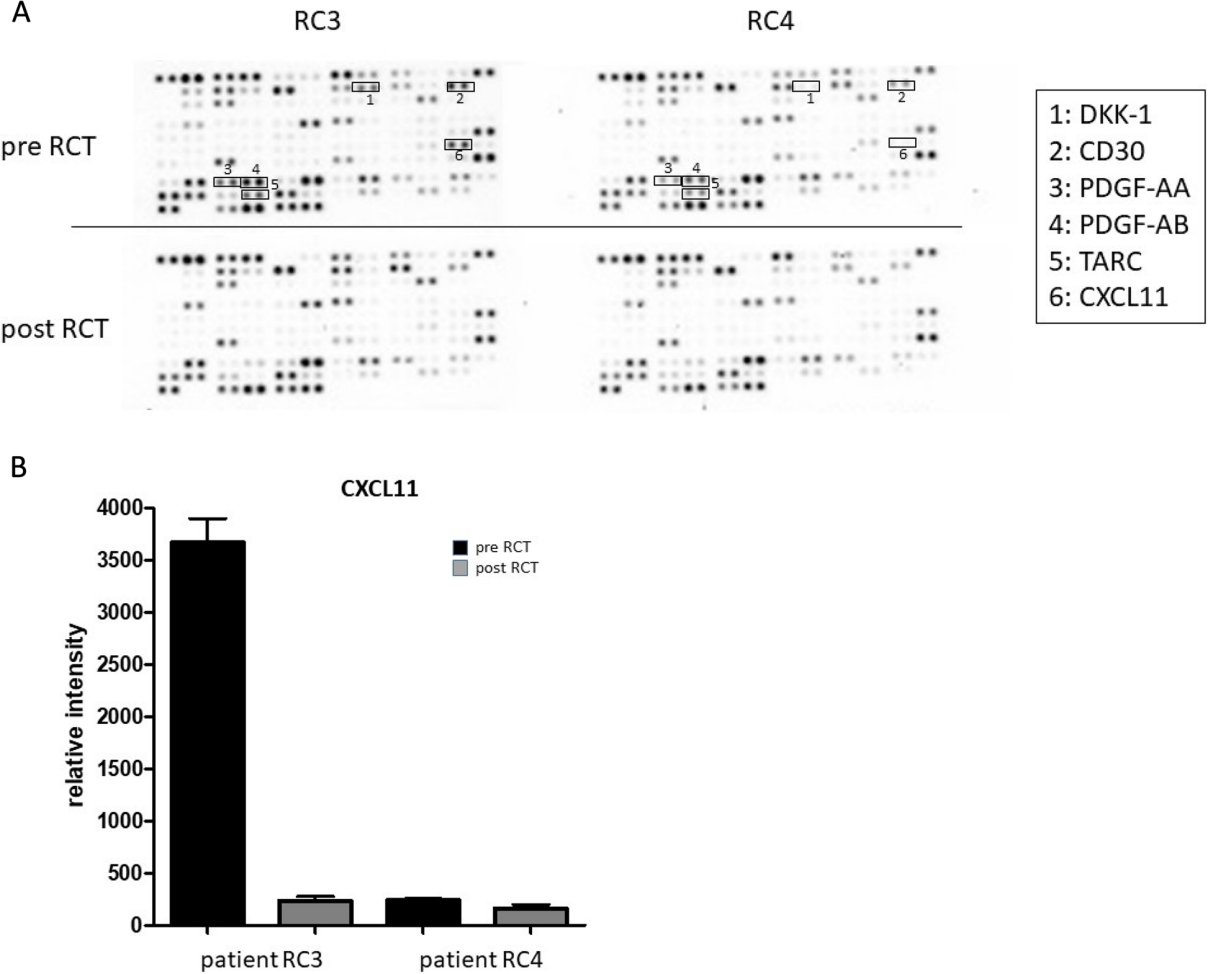
Cytokine screening upon radio(chemo)therapy of HNSCC patients. **A** Raw images of membrane based cytokine arrays of plasma samples of two HNSCC patients (RC3, RC4) before (pre RCT) and after (post RCT) radio(chemo)therapeutic treatment. Decreased expression patterns of certain cytokines (1: BDNF; 2: DKK1; 3: CD30; 4: PDGF-AA; 5: PDGF-AB; 6: TARC; 7: CXCL11) were identified in response to radio(chemo)therapeutic treatment. **B** Semiquantitative analysis was performed by measuring the density of the CXCL11 dots and revealed differential expression levels in the analyzed plasma samples of HNSCC patients RC3 and RC4 before and after radio(chemo)therapeutic treatment

To screen for potential factors which might be responsible for the observed partial recovery of the circulating monocyte subsets, plasma levels of 105 different cytokines and chemokines in two HNSCC patients (RC3, RC4) were measured before and after radio(chemo)therapeutic treatment using a membrane based human cytokine antibody array (Fig. 3A).

Decreased expression patterns of certain cytokines in response to radio(chemo)therapeutic treatment such as DKK1 (Dickkopf WNT Signaling Pathway Inhibitor 1), CD30 (TNF-Rezeptor 8), PDGF-AA/AB (*platelet-derived growth factor)*, TARC (thymus and activation related chemokine), and CXCL11 (C-X-C motif chemokine 11) could be identified (Fig. 3A).

**Fig. 4:**
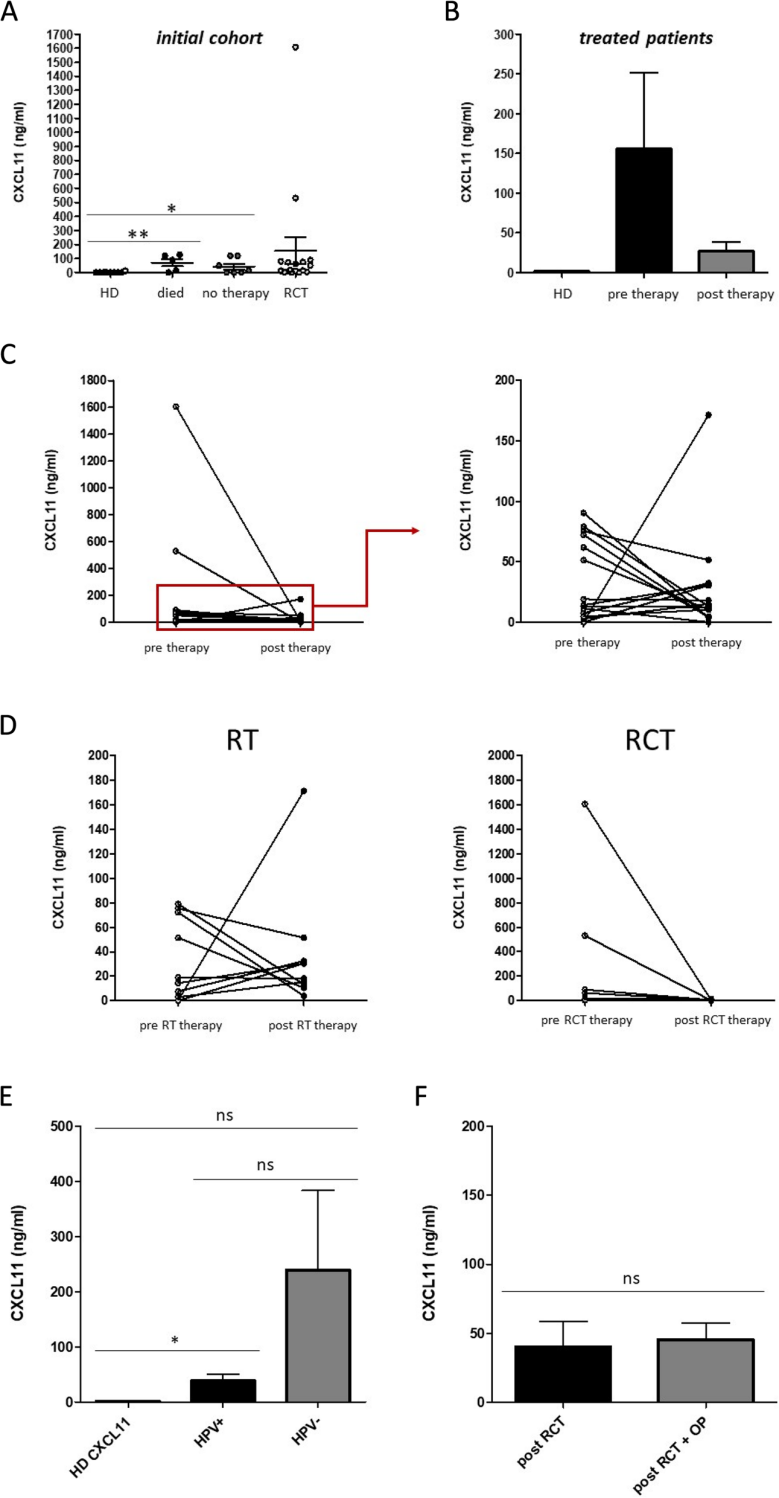
Plasma CXCL11 in HNSCC patients upon radio(chemo)therapy (*n* = 17). **A** ELISA measurements revealed significantly increased CXCL11 levels in patients that unfortunately ‘died’ during the course of therapy (*n* = 5) and in patients that received ‘no therapy’ due to a bad health condition (*n* = 7) compared to healthy donors (*n* = 10). **B** CXCL11 measurements revealed no significant differences between the pre and post therapy situation or healthy donors. **C** Individual plasma CXCL11 pre and post therapy values (ng/ml) revealed decreases as well as increases upon radio(chemo)therapeutic treatment. **D **CXCL11 levels of HNSCC patients that received only radiotherapy (RT) vs. HNSCC patients that received radiochemotherapy (RCT). Our data revealed that radiotherapy resulted in increased CXCL11 levels in some HNSCC patients, whereas radiochemotherapy resulted only in decreased CXCL11 levels. **E** Plasma CXCL11 levels of HPV positive vs. HPV negative HNSCC patients. Data revealed significantly increased CXCL11 levels in HPV + patients compared to healthy donors, but not in HPV negative individuals. **F** Plasma CXCL11 levels of HNSCC patients that received only RTC therapy vs. HNSCC patients that received RTC therapy + surgery. *: *p* < 0.05; **: *p* < 0.01. ns: not significant

Among these factors, chemokine CXCL11 caught our particular interest, because it has recently been shown, that CXCL11 is involved in tumor lymphatic cross talk and the regulation of checkpoint molecule PD-L1 (CD274) in cancer tissues [24, 25]. Semiquantitative analyses were performed by measuring the density of the CXCL11 dots and revealed differential expression levels in the analyzed plasma samples of HNSCC patients RC3 and RC4 before and after radio(chemo)therapeutic treatment (Fig. 3B). In order to quantify the individual plasma CXCL11 levels in our patient cohort, ELISA measurements were performed. Data revealed significantly increased CXCL11 levels in patients who unfortunately died during the course of treatment as well as in patients who received no therapy due to a bad health condition compared to healthy donors (Fig. 4A).

The treatment cohort (RCT) revealed no significant differences to the post therapy situation or healthy donors, due to widely spread measurement values (Fig. 4A, B). A look at the individual plasma CXCL11 pre and post therapy values of our patient cohort revealed decreases as well as increases upon radio(chemo)therapeutic treatment (Fig. 4C). Next, we compared plasma CXCL11 levels of HNSCC patients that received only radiotherapy vs. HNSCC patients that received radiochemotherapy. Our data revealed that radiotherapy resulted in increased CXCL11 levels in some HNSCC patients, whereas radiochemotherapy resulted only in decreased CXCL11 levels (Fig. 4D). Furthermore, we compared plasma CXCL11 levels of HPV positive vs. HPV negative HNSCC patients. Our data revealed significantly increased CXCL11 levels in HPV + patients compared to healthy donors, but not in HPV negative individuals (Fig. 4E). To analyze the potential influence of surgical treatment, plasma CXCL11 levels of HNSCC patients that received only RTC therapy vs. HNSCC patients that received RTC therapy + surgery were compared. Our data revealed no significant differences (Fig. 4F).

**Fig. 5:**
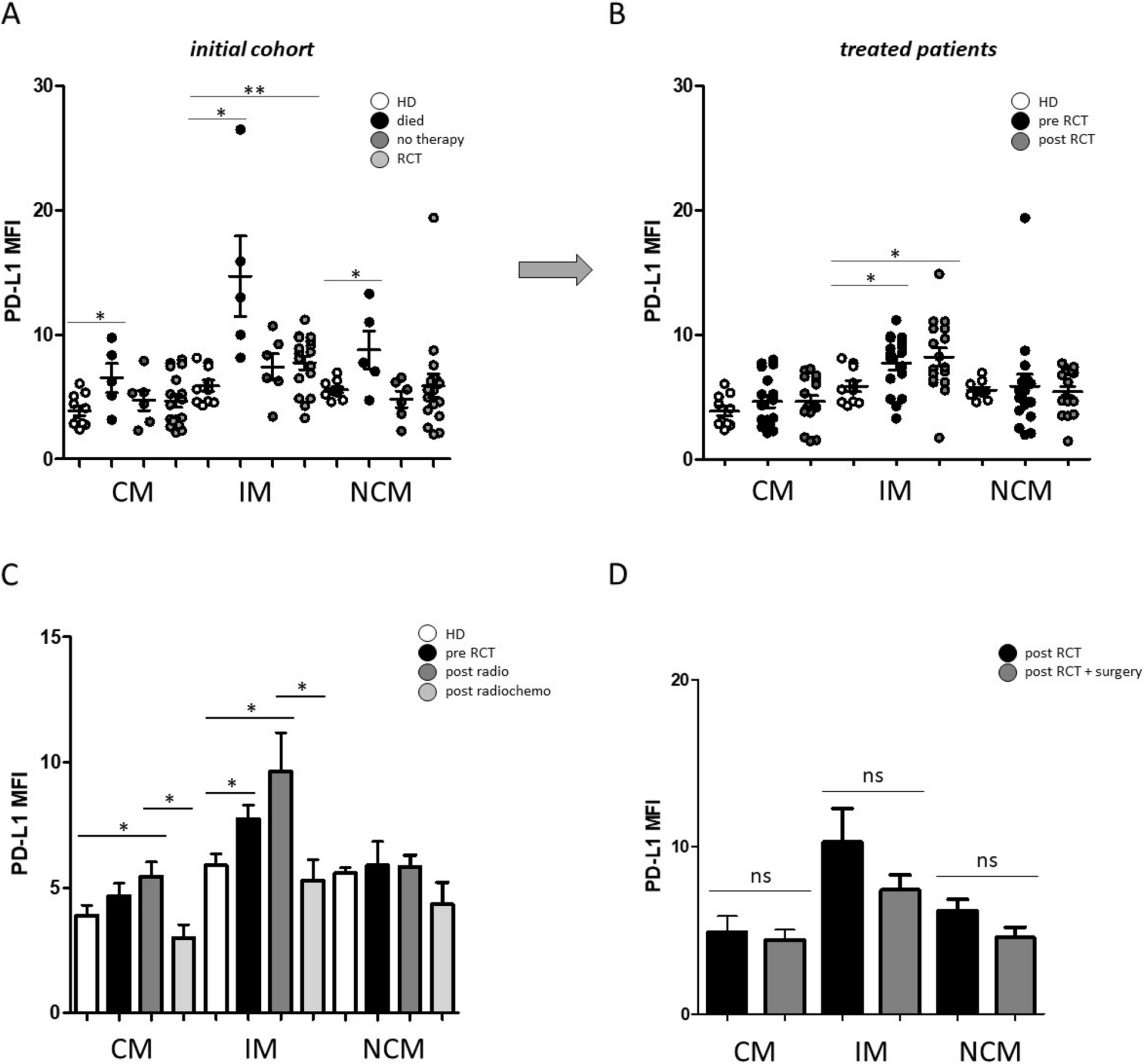
Flow cytometric analysis of PD-L1 on peripheral blood monocyte subsets. **A** PD-L1 expression levels of circulating classical (CM), intermediate (IM) and non-classical monocytes (NCM) in the peripheral blood of HNSCC patients that unfortunately ‘died’ during the course of treatment, patients that received ‘no therapy’ and patients that were able to receive radio(chemo)therapeutic treatment (RCT) compared to healthy donors (HD). **B** PD-L1 expression levels on monocyte subsets of HNSCC patients before (pre RCT) and after (post RCT) radio(chemo)therapeutic treatment compared to healthy donors (HD). **C** PD-L1 expression levels on monocyte subsets of HNSCC patients before RCT and after radio-therapeutic treatment (post radio) and radiochemo-therapeutic treatment (post radiochemo) compared to healthy donors (HD). **D** To analyze the potential influence of surgical treatment, we compared the PDL-1 levels on the different monocyte subsets of HNSCC patients that received only RTC therapy vs. HNSCC patients that received RTC therapy + surgery. *: *p* < 0.05; **: *p* < 0.01; ***: *p* < 0.001. MFI: mean fluorescence intensity. ns: not significant

Due to the potential impact of chemokine CXCL11 on the regulation of checkpoint molecule PD-L1, expression levels on peripheral blood monocyte subsets were analyzed in our initial patient cohort as well as before and after radio(chemo)therapeutic treatment. Data revealed significantly increased PD-L1 on all three monocyte subsets in patients that unfortunately died during the course of treatment compared to healthy donors (Fig. 5A). Moreover, there were significantly increased PD-L1 expression levels on intermediate monocytes in the cohort of treated patients but no significant differences between the pre and post therapy situation (Fig. 5B).

In order to further elucidate these measurements, the cohort of treated patients was further subdivided into patients who received only radiation-therapy (post radio) and patients who received radio- and chemo-therapeutic treatment (post radiochemo). Our data revealed significantly increased PD-L1 expression levels on classical (*p* = 0.0454) and intermediate (*p* = 0.0295) monocytes in response to radiation-therapy. Patients who received radio- and chemo-therapeutic treatment revealed significantly lower PD-L1 expression levels on classical ((*p* = 0.0106) and intermediate (*p* = 0.0471) monocytes compared to only radiation treated patients (Fig. 5). In order to investigate the potential influence of surgery treatment, we compared the PDL-1 levels on the different monocyte subsets of HNSCC patients that received only RTC therapy vs. HNSCC patients that received RTC therapy + surgery (Fig. 5D). Our data revealed no significant differences. An acknowledged limitation of results is the relatively small number of analyzed patients.

**Fig. 6:**
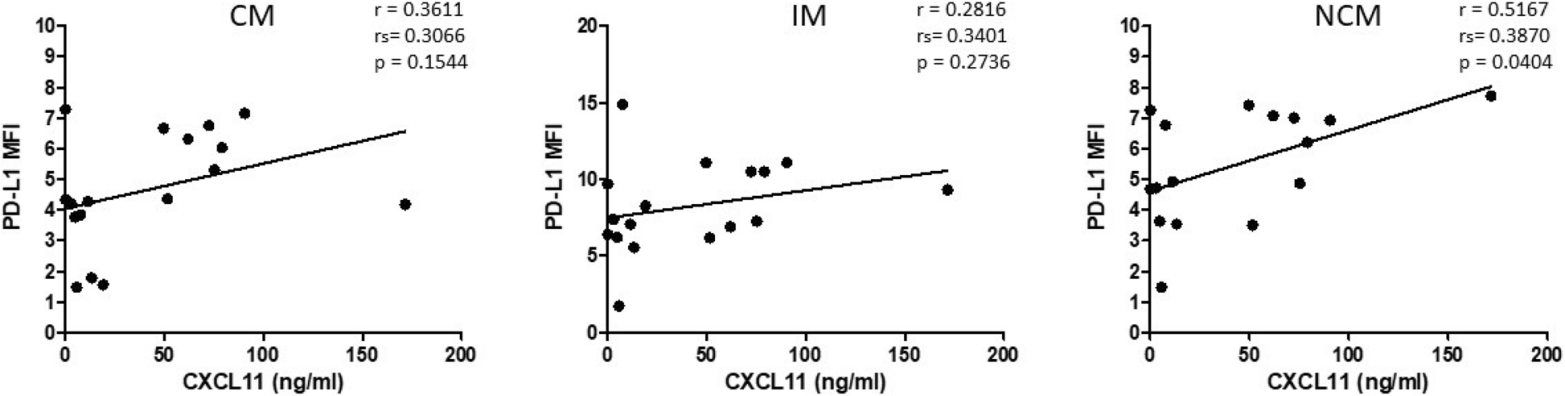
Correlation analysis between the PD-L1 expression of circulating classical (CM), intermediate (IM) and non-classical monocytes (NCM) and plasma levels of chemokine CXCL11 (ng/ml) of HNSCC patients after radio(chemo) therapeutic treatment. The correlation coefficient (r), spearman (rho) correlation coefficient (rs), and p values are given for each correlation. *p* < 0.05 was considered as significant. MFI: mean fluorescence intensity

Next, correlation analyses were performed in order to analyze whether the differential effects of radio(chemo) therapeutic treatment on monocytic PD-L1 and plasma CXCL11 levels of cancer patients might be associated. Data revealed a significant positive correlation (*p* = 0.0404) between plasma CXCL11 and PD-L1 expression on non-classical monocytes after radio(chemo) therapeutic treatment, which indicates an association of these two molecular bioliquid parameters (Fig. 6).

## Discussion

Head and neck squamous cell carcinoma (HNSCC) is one of the most common malignant tumors and standard radio(chemo) therapeutic treatment is applied for over 60% of newly diagnosed cancer patients [26, 27]. It is well known that radio(chemo)therapy exerts a multifaceted systemic immune regulatory influence by inducing the release of inflammatory mediators and immune cells [28]. However, the impact of radio(chemo)therapy on the abundances and cellular characteristics of circulating monocyte subsets in HNSCC is still mostly unclear.

In cancer, monocyte subsets possess diverse activities that contribute to both pro- and anti-tumoral immune response and the dynamics of the subset abundances have been investigated in different studies [29–31]. For instance, increased abundances of classical monocytes were measured during the first 5 cycles of chemotherapy in breast cancer patients, which recovered during the subsequent cycles [32].

In this study, we have shown a partial recovery of peripheral blood monocyte subsets from HNSCC patients upon radio(chemo)therapy compared to the pre-treatment situation. It is well known, that circulating monocytes encounter various tumor derived factors during the time they spend in the blood stream, which affects their behaviors and differentiation [33, 34]. In this context, radio(chemo)therapy can diminish tumor immune escape mechanisms and restore anti-tumor immune response [35–37]. Investigations on the impact of radiation on different blood cell populations revealed that monocytes are more resistant compared to lymphocytes and granulocytes [38].

Our data revealed significantly increased pre-therapeutic expression levels of adhesion molecules CD11a and CX3CR1 on monocyte subsets compared to healthy donors, both of which significantly decreased upon radio(chemo)theraupeutic treatment. Moreover, elevated pre-therapeutic levels of CD11b were measured in circulating monocytes compared to healthy donors.

Integrins CD11a and CD11b are well established leukocyte adhesion molecules, that are expressed on dendritic cells, monocytes and granulocytes and can bind various ligands such as complement factors, collagen or lipopolysaccharide [39, 40]. They are involved in the adhesion of monocytes to the vessel wall and extravasation upon inflammatory processes, such as in patients with coronary artery disease [41]. CX3CR1 is well known to be associated with atherosclerosis and vascular inflammation [42, 43] and is also involved in the monocyte-endothelial interaction and enables leukocytes to crawl along the blood vessels [44]. Elevated levels of CX3CL-1 (CX3C-chemokine ligand 1) receptor CX3CR1 are distinctive markers for CD16^+^ monocytes in both men and mice [44]. These data corroborate different studies that revealed a reduced recruitment of peripheral blood monocytes due to an attenuated chemotactic activity of tumor cells upon therapeutic treatment. It has been shown that radiation as well as chemotherapy modify the migratory ability of monocytes in a drug- and cell-type-specific manner via systemic alteration of chemoattractant factors [45–48].

Next, measurements of pre- and post therapy plasma levels of different cytokines and chemokines revealed decreased expression patterns of certain cytokines such as DKK1 (Dickkopf WNT Signaling Pathway Inhibitor 1), CD30 (TNF-Rezeptor 8), TARC (thymus and activation related chemokine), and PDGF-AA/AB (*platelet-derived growth factor) in response to therapeutic treatment*.

DKK1 is an important factor of the immunosuppressive tumor microenvironment in head and neck squamous cell carcinoma and parcticipates in the development of resistance to radiotherapy and immunotherapy [49].

Elevated pre-therapeutic plasma levels of CD30 and TARC have also been reported in patients with classical Hodgkin lymphoma and decreased upon treatment [50]. Furthermore, platelet-derived growth factors have been shown to be correlated with tumorigenensis and poor prognosis in oral squamous cell carcinoma and decreased levels were observed in response to chemotherapy [51, 52]. However, it has recently been shown that PDGF does not act as a monocyte chemoattractant [53].

Of note, plasma levels of chemokine CXCL11 (C-X-C motif chemokine 11) were found to be decreased in the majorioty of HNSCC patients upon radio(chemo)therapy, but also increased values were identified in some patients. It has been shown in colorectal cancer that decreased CXCL11 levels were associated with an inhibition of cancer cell growth and epithelial-mesenchymal transition [54]. Elevated CXCL11 also increases the aggressiveness of breast cancer cells [55]. Furthermore, CXCL11 is involved in tumor lymphatic cross talk and the regualtion of checkpoint molecule PD-L1 (CD274) in cancer tissues [24, 25]. In contrast, it has recently been shown in glioblastoma, that CXCL11 had a potent antitumor effect and reprogrammed the immunosuppressive tumormicroenvironment [56]. We further compared plasma CXCL11 levels of HNSCC patients that received only radiotherapy vs. HNSCC patients that received radiochemotherapy. Our data revealed that radiotherapy resulted in increased CXCL11 levels in some HNSCC patients, whereas radiochemotherapy resulted only in decreased CXCL11 levels. These data go along with another study, where it has been shown that radiotherapy stimulates tumor cells and stromal cells to produce chemokines, such as CXCL9, CXCL10, CXCL11 and CXCL16, which lead to the infiltration of DCs, macrophages and T cells, further promoting inflammatory tumor microenvironment. In an actual publication, it has been shown in glioblastoma, that CXCL11 had a potent antitumor effect and reprogrammed the immunosuppressive tumor microenvironment, in which increased infiltration of CD8 T cells, NK cells and M1 macrophages, but decreased abundances of myeloid-derived suppressor cells (MDSCs), regulatory T cells (Tregs) and M2-polarized macrophages were observed [57]. Furthermore, we compared the plasma CXCL11 levels of HPV positive vs. HPV negative HNSCC patients. Our data revealed significantly increased CXCL11 levels in HPV + patients compared to healthy donors, but not in HPV negative individuals. Despite the relatively small sample size of our cohort, these data are go along with an actual publication which has recently shown that antiviral treatment significantly reduces the levels of plasma CXCL11 [58].

Checkpoint molecule PD-L1 is involved in the immune regulation of different immune cells and the interaction of PD-1/PD-L1 attenuates immune responses and thus supports tumor immune escape mechanisms [59–62]. PD-L1 is known to be involved in different aspects of immune regulation and expressed in different types of immune cells including B cells, T cells, dendritic cells and monocytes [63–66]. Myeloid cells in particular are important regulators of cancer development and progression, and PD-L1 expression on myeloid cells has been correlated with poor prognosis of tumor patients [67–69]. PD-L1 overexpression on monocytes was found to promote cancer progression in lung adenocarcinoma [70].

With regard to monocytic PD-L1, we further subdivided our cohort into patients who received only radiation-therapy and patients who received radio- and chemo-therapy. Data revealed significantly increased PD-L1 expression levels on classical and intermediate monocytes in response to radiation-therapy, but significantly decreased PD-L1 expression levels upon radio- and chemo-therapeutic treatment. The population of peripheral blood monocytes is renewed about every 5 days and it is well known that chemo-therapy induces the proliferation of progenitors and the subsequent entry of naïve monocytes into the blood stream, which are less influenced by the tumor microenvironment [36, 37, 71] and therefore might reveal a normal PD-L1 expression level. It has been shown in different studies, that radiotherapy can affect the entire tumor microenvironment and might induce the expression immunosuppressive molecules PD-L1 [72].

Correlation analyses revealed a significant correlation between plasma CXCL11 levels and PD-L1 expression on non-classical monocytes, which corroborates earlier studies that have shown the involvement of CXCL11 in the regulation of checkpoint molecule PD-L1 in human cancers [24, 25]. In summary, our study suggests a partial recovery of circulating monocytes in HNSCC patients upon radio(chemo)therapeutic treatment, with differential effects of the individual therapy regimen. Significant correlations were identified between PD-L1 expression on peripheral blood monocytes and plasma CXCL11 levels, both of which could provide helpful information concerning therapy response and the individual immunologic situation. An acknowledged limitation of our study is the relatively small number of patients. Further comprehensive investigations on larger patient cohorts in correlation with the specific individual therapy regimen, therapy response, and patient survival are required to elucidate the meaningfulness of peripheral blood monocyte subsets and chemokine CXCL11 as potential bioliquid indicators in HNSCC.

## Materials and methods

### Ethics statement

All patients were treated at the Department of Otorhinolaryngology, University Hospital Schleswig-Holstein, Campus Luebeck, and have given their written informed consent. The study was approved by the local ethics committee of the University of Luebeck (approval number 16–278) and conducted in accordance with the ethical principles for medical research formulated in the WMA Declaration of Helsinki.

### Tumor material, blood collection and patient data

We evaluated a cohort of 29 HNSCC patients prior and post radio(chemo)therapy in terms of the peripheral blood monocyte subset distribution and expression of PD-L1 and different monocytic adhesion molecules. The patients were diagnosed in the Institute for Pathology at the University Hospital Schleswig-Holstein in Luebeck. The clinical data of the HNSCC patients were obtained from clinical and pathological records and afterward anonymized. TNM stages were assessed by the 8th edition of the TNM classification for HNSCC. All blood donors have signed a written consent, and were informed about the aims of the study and the use of their samples. Blood was drawn by venipuncture into a sodium citrate containing S-Monovette (Sarstedt; Nümbrecht, Germany). Blood samples were collected from healthy donors (*n* = 10; 6 female/4 male; mean age of 59) from HNSCC patients (*n* = 29; mean age of 67). The clinicopathological characteristics of the patients are listed in Table 1.

**Table 1:** Clinicopathological parameters

Characteristics	Patients (*n*=29)	
	*n*	%
**Gender**		
M	18	72
F	11	28
**Tumor Site**		
Pharynx	15	51
Larynx	5	17
Oral cavity	9	32
**Tumor Stage**		
T1 - T2	13	64
T3 -T4	16	36
**HPV status**		
Positive	11	38
Negative	11	38
Unknown	7	24
**Alcohol abuse**		
Yes	5	17
No	24	83
**Tobacco consumption**		
Yes	21	72
No	8	28

### Staining of monocyte subsets in whole blood

Within 4 h after blood collection, 20 µl of citrate blood was diluted in 80 µl PBS. Blood cells were stained with following antibodies: CD45-PE, CD14-FITC, CD16-BV-510, HLA-DR-APC-Cy7, CX3CR1-BV421, CD11b-BV421 and CD3-PerCP (all from Biolegend, San Diego, USA). After 25 min staining in the dark, 650 µl RBC Lysis Buffer (Biolegend) were added to the samples and incubated for another 20 min. Subsequently, suspension was centrifuged at 400 x g for 5 min and supernatant was discarded. Cell pellet was resuspended in 100 µl fresh PBS and used for FACS analysis.

### FACS analysis

Flow cytometry was performed with a MACSQuant 10 flow cytometer (Miltenyi Biotec, Bergisch-Gladbach, Germany) and data were analyzed using the FlowJo software version 10.0 (FlowJo, LLC, Ashland, USA). All antibody titrations and compensations were performed in beforehand. For whole blood measurements, at least 100 000 CD45^+^ leukocytes were analyzed. Gating of monocyte subsets was performed as described before [23].

### Cytokine analysis

To determine plasma cytokine expression patterns upon radio(chemo)therapeutic treatment of HNSCC patients, cytokine arrays were performed. Supernatants from cell cultures were collected after incubation and instantly frozen with liquid nitrogen and preserved at -80 °C. Proteome Profiler^™^ Human XL cytokine arrays (R&D Systems, Minneapolis, MN, USA) were hybridized with the cell culture medium as recommended by the supplier. Expression was visualized using an enhanced chemiluminescence detection kit (R&D Systems, Minneapolis, United States). Semiquantitative analysis was performed by measuring the density of the bands using an iBright CL 1000 biomolecular imager (Invitrogen, Carlsbad, CA, USA).

Plasma concentrations of chemokine CXCL11 were assessed from citrate-plasma samples and were determined by enzyme-linked immunosorbent assays (ELISA) according to manufacturer’s protocols (R&D Systems, Minneapolis, MN, USA).

### Statistical analysis

Statistical analyses were performed with GraphPad Prism Version 7.0 f. The mean and standard error (SEM) are presented. The differences between groups were determined after testing for normal distribution and applying parametric (student`s t-Test), or non − parametric 1-way Anova with Bonferroni post hoc test. The correlation between parameters was calculated using multivariate regression with the Pearson correlation coefficient and the spearman (rho) correlation coefficient. *p* < 0.05 (*), *p* < 0.01 (**), and *p* < 0.001 (***). Additional statistical details are given in the respective figure legends, when appropriate.

## Data Availability

No datasets were generated or analysed during the current study.
